# Six-hour central venous oxygen saturation has no prognostic value in patients with septic shock

**DOI:** 10.1186/2197-425X-3-S1-A220

**Published:** 2015-10-01

**Authors:** KM Yoo, KS Kim, GJ Suh, WY Kwon, JS Kim, MJ Park, YJ Choi, K Kim

**Affiliations:** Seoul National University Hospital, Department of Emergency Medicine, Seoul, Korea Republic of Korea; Seoul National University Bundang Hospital, Department of Emergency Medicine, Seongnam-si, Korea Republic of Korea

## Intr

Central venous oxygen saturation (Scv02) is used as an indicator of adequate tissue oxygenation and current sepsis guideline includes Scv02 70% or more as one of initial resuscitation goals during the first 6 hours. However, it is still controversial that to achieve Scv02 goal is mandatory.

## Objectives

The aim of this study was to investigate the prognostic value of 6-hour Scv02 to predict the 1-month mortality in patients with septic shock.

## Methods

We have retrospectively identified septic shock patients who received protocolized treatment in two tertiary academic EDs. All patients were treated using the early goal-directed protocol. The data with respect to demographics, predisposing factors, site of infection, and the admission APACHE II score were collected. Hemodynamic (mean arterial pressure and central venous pressure) and laboratory (arterial blood gas analysis, Scv02, and lactate level) parameters at baseline and 6-hour were also recorded. To test the prognostic value of 6-hour Scv02, the area under receiver operating characteristics curve (AUROC) to predict 1-month mortality was calculated and compared with that of 6-hour lactate level. Pearson correlation coefficient between 6-hour Scv02 or lactate level and the admission APACHE II score were also analyzed.

## Results

**Figure 1:**
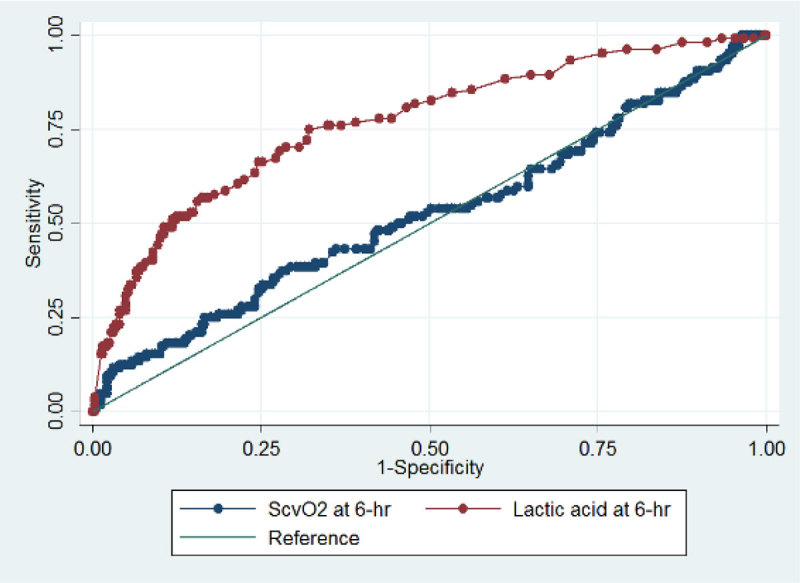
**The 6-hour lactate level was positively correlated with the admission APACHE II score (Pearson´s ρ = 0.556). However, the 6-hour Scv02 was not correlated with the admission APACHE II score (Pearson´s ρ = -0.022) (Fig 2).**

After excluding 122 patients with missing variables, 424 were analyzed. Among them, 104 (24.5%) died within 1-month. Respiratory infection was more frequently observed in 1-month mortality group. Lower 6-hour mean arterial pressure, higher baseline and 6-hour lactate levels, and higher admission APACHE II score were associated with an increase in the 1-month mortality. The AUROC of 6-hour Scv02 to predict 1-month survival was 0.525 (95% confidence interval, 0.458-0.592). AUROC of 6-hour lactate level to predict 1-month mortality was 0.767 (0.713-0.821), which was significantly higher than that of 6-hour Scv02 (p < 0.001) (Figure 1).

**Figure:**
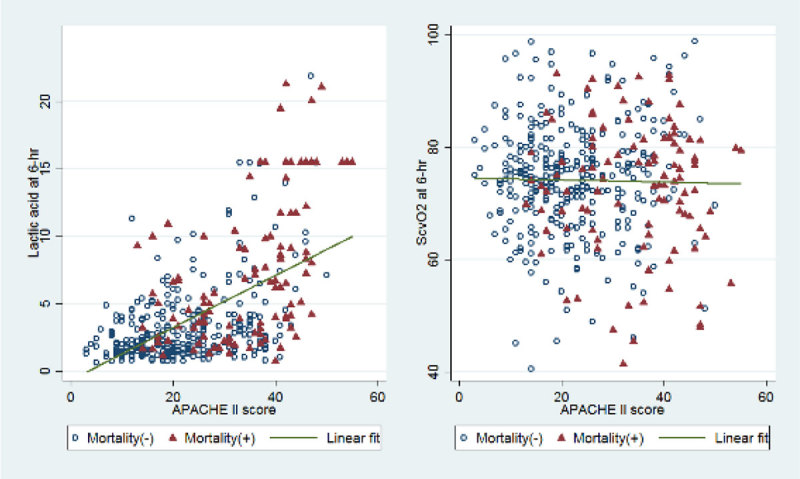
Figure 2

## Conclusions

The 6-hr Scv02 has no prognostic value in patients with septic shock. Targetting Scv02 goal ≥ 70% may not be essential in the management of septic shock.
